# THE MRI-COMPATIBLE STIMULATION SYSTEM TO CHARACTERIZE THE SUPRASPINAL CONTROL OF GAIT IN AGING: A SYSTEMATIC REVIEW

**DOI:** 10.1093/geroni/igad104.0269

**Published:** 2023-12-21

## Abstract

The regulation of gait depends upon both peripheral and supraspinal elements. The characterization of these elements can provide insights into how aging and age-related conditions affect gait performance in older adults. Functional magnetic resonance imaging (fMRI) is one widely-used technique to sophisticatedly measure the supraspinal elements. However, people are required to stay motionless during MRI scan, it is thus challenging to characterize the supraspinal control of gait. To overcome this limitation, multiple MRI-compatible devices have been developed to apply the walking-related sensorimotor stimuli. Still, large variance in device design and observation exists, limiting their research applications. We here thus performed a thorough systematic review to comprehensively summarize the progress of this field. Seven electronic databases (e.g., PubMed) were searched. The information on device design, study protocol and neuroimaging observations of twenty-six studies using 13 types of devices was extracted. Specifically, three of these devices can induce somatosensory-only stimuli, eight for motion-only, and two for both types of stimuli. The results demonstrated that using these devices can successfully obtain valid and reliable fMRI data when participants experiencing sensorimotor stimulation. Multiple supraspinal regions, such as the sensorimotor regions, prefrontal cognitive and attention regions, and cerebellum were activated by the sensorimotor stimuli. The evidence level of these observations was still low due to small sample size of participants who were not explicitly screened. It is thus needed to further enhance the performance of these devices and to examine and confirm the findings via studies with more rigorous design (e.g., larger participant sample size).

